# A cross-sectional study on associations of physical symptoms, health self-efficacy, and suicidal ideation among Chinese hospitalized cancer patients

**DOI:** 10.1186/s12888-020-02945-x

**Published:** 2020-11-19

**Authors:** Qingyi Xu, Shuhua Jia, Maiko Fukasawa, Lin Lin, Jun Na, Zhen Mu, Bo Li, Ningning Li, Tong Zhao, Zaishuang Ju, Meng He, Lianzheng Yu, Norito Kawakami, Yuejin Li, Chao Jiang

**Affiliations:** 1 Department of Psychology, College of Humanities and Social Sciences, Dalian Medical University, Dalian, China; 2 Department of Mental Health, the Graduate School of Medicine, the University of Tokyo, Tokyo, Japan; 3 School of Psychology, Tianjin Normal University, Tianjin, China; 4 Liaoning Provincial Center for Disease Control and Prevention, Institute of Chronic Disease, Shenyang, China; 5 Department of Geriatrics, Guangzhou Brain Hospital, Guangzhou, China; 6 Department of Psychology, Benxi Kangning Hospital, Benxi, China; 7 Department of Oncology, Zhongshan Affiliated Hospital of Dalian University, Dalian, China; 8 Department of Oncology, Second Affiliated Hospital of Dalian Medical University, Dalian, China; 9 Department of Biology, Morgan State University, Baltimore, Maryland USA

**Keywords:** Suicidal ideation, Cancer, Physical symptoms, Self-efficacy

## Abstract

**Background:**

Epidemiological studies have shown increased risk of suicide in cancer patients compared with the general population. The present study aimed to examine the association between physical symptoms and suicidal ideation in Chinese hospitalized cancer patients and test the modifying effect of health self-efficacy on the association.

**Methods:**

A cross-sectional study was conducted with 544 hospitalized cancer patients in two general hospitals in northeast China via face-to-face interviews. Suicidal ideation was measured by using the first four items on the Yale Evaluation of Suicidality scale and then dichotomized into a positive and negative score. Multivariate logistic regression analyses were conducted to examine the impacts of physical symptoms, health self-efficacy, and their interactions on suicidal ideation.

**Results:**

The suicidal ideation rate was 26.3% in the enrolled cancer patients. Logistic regression showed that insomnia (aOR = 1.84, 95% CI 1.13 to 3.00, *p* = 0.015) and lack of appetite (aOR = 2.14, 95% CI 1.26 to 3.64, *p* = 0.005) were significantly associated with suicidal ideation. Low health self-efficacy had a marginally significant exaggerating effect on the association between pain and suicidal ideation (aOR = 2.77, 95% CI 0.99 to 7.74, *p* = 0.053), after adjusting for significant socio-demographics, clinical characteristics, and depression.

**Conclusions:**

These findings demonstrate significant associations between physical symptoms (insomnia and/or lack of appetite) and suicidal ideation and highlight the potential modifying role of health self-efficacy in the identification and prevention of suicide among cancer patients.

## Background

Cancer is a leading cause of death worldwide [1]. The diagnosis of cancer and the subsequent treatments may evoke psychological and physical distresses in patients, which affects life quality and even the will to live [2–4]. Suicide rate among cancer patients is generally twice as high as that of the general population [5, 6]. Prior suicidal ideation (SI) is one of the primary risk factors for future suicidal behaviors [7, 8] and 60 % of the first suicide attempts occur within a year of SI onset [9]. Therefore, early detection of SI in cancer patients is critical for reducing the incidence of suicide and suicide attempts.

It has been reported that up to 71% of cancer patients have reported SI in clinical settings [10]. The possible risk factors for SI include female gender [11], old age [12], unmarried status [13, 14], retirement [15], recurrence [2], cancer metastasis [2, 16], mental disorders [17] and pain [18–20]. Mental illnesses such as depressive disorders are known to be a significant predictor for SI [4, 15, 16]. However, it is very challenging for oncologists to diagnose depression due to the complexities involved in conducting depression-related clinical interviews. Additionally, although SI is a diagnostic criterion of depression, depression is not an essential precondition for SI to occur [21]; in other words, one may have SI without any diagnosable depression. Physical complaints such as insomnia and appetite loss that are commonly observed in patients with cancer [22] also serve as diagnostic criteria for several mental disorders, e.g. depressive disorders. Therefore, it is of great interest to investigate whether such physical symptoms increase the risk of SI in both populations with or without depressive disorders.

There has been growing evidence that persistent poor physical status significantly increases the risk of suicidal thoughts in cancer patients [4, 18, 22–24]. A study on 48 cancer patients with metastasis revealed that the burden of physical symptoms is more correlated with desire for death than pain or depression [22]. However, the effect of physical status on SI has drawn relatively little attention compared with other psychiatric symptoms. Most studies either analyzed the association between numbers/sum-score of physical symptoms and SI or just focused on pain as the main risk factor [11, 18, 19, 25]. To the best of our knowledge, so far, there has been only one study that investigated the association between individual physical symptoms (i.e. diarrhea, hair loss, usual fatigue) and SI, however, this study only focused on patients with stomach cancer in South Korea [23]. An analysis of specific symptoms offers opportunities to identify physical indicators for SI whereas prior studies summing symptoms may miss important information [26]. Therefore, it is valuable to investigate whether each specific physical symptom is associated with SI, which would improve early detection of the potential suicide risk in cancer patients.

Expressions of depression vary across different cultures [27]. It has been reported that Asians express more physical symptoms than Westerners when experiencing depression, while the latter prone to express cognitive and psychological symptoms [27, 28]. Thus, the association between physical symptoms and SI may be different in Asian countries, e.g. China, from that of western countries. However, there is a paucity of such studies on cancer patients in China.

Health self-efficacy, referring to individual’s perceived ability to cope with stress and their self-confidence in overcoming challenges to their health [29, 30], has been demonstrated to play a critical role in pain management and health adjustment [31]. A previous study found that greater illness burden was significantly associated with lower health self-efficacy [32]. Moreover, emerging evidence suggests that low health self-efficacy increases the risk of suicide behaviors over the course from prior ideation to future intention [33, 34]. Therefore, health self-efficacy may have a modifying effect on the association between physical symptoms and SI. However, to the best of our knowledge, few studies have examined the potential modifying effect.

The present study aimed to examine the association between physical symptoms and SI in Chinese hospitalized cancer patients, and the potential modifying effect of health self-efficacy on this association. We hypothesized that: 1) physical symptoms are associated with an increased risk for SI, even after controlling for established predictors; 2) the association between physical symptoms and SI is greater for patients with low levels of health self-efficacy, compared to those with high levels.

## Methods

### Participants and procedures

The cross-sectional study was conducted in two general hospitals (the Second Affiliated Hospital of Dalian Medical University and the Zhongshan Affiliated Hospital of Dalian University) in Dalian, a metropolitan region located on the northeast coast of China. Dalian’s gross domestic product (GDP) ranked 26th nationally in 2018, falling into the medium level among all the cities in China. Patients who were hospitalized in the oncology settings at the two hospitals were consecutively recruited from January to December 2015. Eligibility criteria included 1) diagnosis of cancer by pathology or cytology; 2) age of 18 years or older; 3) patient awareness of their own cancer diagnosis; 4) provision of written informed consent. Exclusion criteria were 1) inability to participate in the survey independently due to serious illness, cognitive impairment (such as dementia or delusions) or other medical conditions; 2) language communication barriers or lack of capability to cooperate in the interview; 3) family caregivers who required confidentiality of the cancer diagnosis from the patient. All the procedures were approved by the Committee on Human Ethics of Dalian Medical University. All enrolled patients signed the written informed consent.

Trained research staff conducted the interview after the patients had undergone at least 3 days of clinical treatments. We asked whether they were bothered by each of fourteen physical symptoms over the previous 2 days in the interview. The face-to-face interviews lasted 40–50 min. All the measures were verbally administered to enhance data accuracy and reduce the frequency of missing data. Cancer-related clinical information, i.e. type of cancer, stage at diagnosis, medical treatment, and metastatic status, were extracted from the patients’ medical records.

### Measures

#### Suicidal ideation

The Yale Evaluation of Suicidality scale is a 16-item structured questionnaire that assesses current suicidal thoughts and actions, history of suicide attempts, and feelings/attitudes on suicide [35]. It has demonstrated adequate validity in cancer patients [3, 35] (see Additional file 1). The first four items in the questionnaire compose a screening measure that assesses the strength of the patients’ wish to live and wish to die (passive SI), the existence of thoughts of killing himself/herself (active SI), and the weighing of dying over living. Because of the rarity with which any suicidality was endorsed, all four screening items were used to assess SI in this study (Cronbach’sα = 0.93). Aiming to detect both clear and potential suicidal thoughts, we dichotomized the patients’ scores, where a positive screen (endorsement of any of the four items) =1 and a negative screen = 0.

#### Independent variables and covariates

Physical symptoms were measured with one item from the McGill Quality of Life Questionnaire (MQOL) [36]. Participants were asked whether they were bothered by each of 14 symptoms over the previous 2 days with a response of yes and no. Fourteen items were included: pain, shortness of breath, insomnia, weakness, fatigue, nausea, lack of appetite, constipation, diarrhea, edema, cough, vomiting, fever, and bloating.

#### Health self-efficacy

We measured health self-efficacy by using the Chinese version of the Strategies Used by People to Promote Health (SUPPH) [37], a 29-item validated scale including three dimensions. Each item is rated on a five-point scale ranging from “little confidence” =1 to “quite a lot of confidence” =5. All the items were summed into a score with a higher score indicating a higher sense of health self-efficacy. To evaluate the relative risk of SI, the scores were categorized into high (≥50) and low groups (< 50) by the median. The Cronbach’s α of the present data was 0.96.

#### Depression

Clinical diagnosis of depression was evaluated by the 17-item version of the Hamilton Depression Rating Scale (HDRS-17) that assesses the severity of depression symptoms over the past week [38]. It contains 17 items to detect depressive disorders and identifies the severity classification by semi-structured interview. A higher total score indicates a greater severity of depression. Severity classifications are defined as follows: 0–7 for no depression, 8–16 for mild depression, 17–23 for moderate depression, and ≥ 24 for severe depression. According to the proposal by Zimmerman et al. [39], we used the cut-off value of 8 to identify depressive disorders, which showed satisfying reliability (Cronbach’s α = 0.84; Split reliability = 0.79).

#### Other variables

Socio-demographic characteristics included sex, age, marital status, education, residence, employment status, household income, smoking, and drinking. Cancer-related clinical variables included cancer type, stage at diagnosis, medical treatment, time since diagnosis, and metastasis status.

### Statistical analysis

Descriptive analyses and chi-square were used to compare the socio-demographics and clinical variables between the cancer patients with and without SI. Multivariate logistic regression was conducted to determine the associations between physical symptoms and SI with three models.

We also tested the interaction of health self-efficacy and each physical symptom on SI using multivariate regression analysis. The ratios were adjusted for potential confounding factors to SI, which included socio-demographics, clinical characteristics, and diagnosed depression. All statistical analyses were performed by using IBM SPSS version 21.0. The reported CIs were calculated at the 95% and the statistical significance was set at 0.05 level. All tests were two-sided.

## Results

### Sample characteristics

A total of 700 cancer patients were contacted for this study. Among them, 59 eligible patients refused to join and 69 patients were not able to cooperate due to at least one of the following factors: poor physical conditions, poor mental health conditions, and low education level. Initially, 282 out of 350 eligible hospitalized patients from Zhongshan Affiliated hospital of Dalian University (response rate 80.6%), and 290 out of 350 from Second hospital of Dalian Medical University (response rate 82.9%) agreed to participate in the study, however, 28 of them failed to complete the questionnaire and dropped out in the halfway. As a result, a total of 544 patients were included in the analysis (valid rate 95.1%). The sample characteristics are displayed in Table 1. The sample consisted of both male (49.2%) and female (51.8%) patients, approximately male:female = 1:1, at average age of 59.9 years (range,19–81 years old; standard deviation, 11.6 years). The majority (78.3%) primarily lived in the cities. Over 70% of the participants were clustered into three types of cancers: digestive system cancer (29%), lung cancer (23.7%), breast cancer (18.9%). The rest were diagnosed with gynecological cancers (9.6%), brain and neck cancers (6.4%), leukemia/lymphoma (5.9%), and others (6.4%). About half of the participants had metastasis and diagnosed with cancer for more than 1 year. The percentage of missing data was less than 1.5% for all variables and missing data on all variables except for SI was assumed to be in the low-risk category.

**Table 1:** Characteristics of the sample (*N* = 544)

	N	%
Sex		
Men	262	48.2
Women	282	51.8
Age (years)		
18–49	82	15.1
50–64	282	51.8
65+	180	33.1
Marital status		
Married	433	79.6
Separated, divorced, bereaved	85	15.6
Never married	26	4.8
Education		
Less than high school	269	49.4
High school	143	26.3
College or higher	132	24.3
Residence		
City	426	78.3
Rural	118	21.7
Employment status		
Employed	99	18.2
Unemployed	47	8.6
Retired	343	63.1
Farm work	50	9.2
Other	5	0.9
Cancer diagnosis		
Digestive tract	158	29.0
Lung	129	23.7
Breast	103	18.9
Gynecologic	52	9.6
Brain and Neck	35	6.4
Leukemia/Lymphoma	32	5.9
Other	35	6.4
Stage at diagnosis		
I	52	9.6
II	108	19.9
III	163	30.0
IV	217	39.9
Unknown	4	0.7
Time since diagnosis		
Less than 3 months	123	22.6
4–12 months	148	27.2
13–36 months	156	28.7
More than 36 months	117	21.5
Metastasis present	296	54.4

### Prevalence of suicidal ideation

Out of the total participants (*n* = 544), over a quarter of them reported that they had experienced SI. The prevalence of SI was 26.3% (*n* = 143), with 24.4% in men and 28.0% in women.

### Study factors associated with suicidal ideation

The comparison of socio-demographic factors and the related clinical characteristics between the enrolled patients with and without SI is displayed in Table 2. We found no significant difference in the following demographic variables: sex, age, education attainment, or household income between the two groups (all *p* > 0.05). A significant difference was found with marital status, employment status, metastasis, and currently diagnosed depression (all *p* < 0.05). The comparison of the stage at diagnosis showed a marginal *p* value (*p* = 0.051). In addition, the two groups of patients presented significantly different levels of health self-efficacy and profiles of physical symptoms, in particular, symptoms like pain, shortness of breath, insomnia, nausea, and lack of appetite. Based on these results, we entered socio-demographic factors, metastasis, stage at diagnosis, and depression as control variables in the subsequent analyses.

**Table 2:** Characteristics of Cancer patients with and without Suicidal Ideation (*N =* 544)

	Without Suicidal		With Suicidal		t/χ^2^	*p*
	Ideation (*n* = 401)		Ideation (*n =* 143)			
	n	%	n	%		
Sex					0.90	0.302
Men	198	75.6	64	24.4		
Women	203	72.0	79	28.0		
Age (years)					4.26	0.119
18–49	68	82.9	14	17.1		
50–64	204	72.3	78	27.7		
65+	129	71.7	51	28.3		
Marital status					7.33	**0.026**
Married	330	76.2	103	23.8		
Separated, divorced, bereaved	53	62.4	32	37.6		
Never married	18	69.2	8	30.8		
Education					1.22	0.543
Less than High school	194	72.1	75	27.9		
High school	105	73.4	38	26.6		
College or higher	102	77.3	30	22.7		
Residence					3.59	0.058
City	306	71.8	120	28.2		
Rural	95	80.5	23	19.5		
Employment status					12.51	**0.014**
Employed	85	85.9	14	14.1		
Unemployed	36	76.6	11	23.4		
Retired	242	70.6	101	29.4		
Farm work	36	72.0	14	28.0		
Other	2	40.0	3	60.0		
Household income monthly					2.14	0.342
≤ 3000	102	71.3	41	28.7		
3000–5000	125	71.4	50	28.6		
5000+	174	77.0	52	23.0		
Smoking habits					0.09	0.764
No	258	74.1	90	25.9		
Yes	143	73.0	53	27.0		
Drinking habits					1.40	0.237
No	116	77.3	34	22.7		
Yes	285	72.3	109	27.7		
Cancer diagnosis					5.40	0.494
Digestive tract	116	73.4	42	26.6		
Lung	98	76.0	31	24.0		
Breast	75	72.8	28	27.2		
Gynecologic	39	75.0	13	25.0		
Brain and Neck	21	60.0	14	40.0		
Leukemia/Lymphoma	23	71.9	9	28.1		
Other	29	82.9	6	17.1		
Stage of cancer					9.44	0.051
I	39	75.0	13	25.0		
II	78	72.2	30	27.8		
III	132	81.0	31	19.0		
IV	148	68.2	69	31.8		
Unknown	4	100.0	0	0.0		
Medical treatment, ever					9.03	0.108
Surgery only	40	81.6	9	18.4		
Radion & Surgery + Radion	11	52.4	10	47.6		
Chemo & Surgery+Chemo	297	75.2	98	24.8		
Chemo+ Radion	12	66.7	6	33.3		
Surgery+Chemo+ Radion	33	68.8	15	31.3		
None of above	8	61.5	5	38.5		
Metastasis					8.83	**0.003**
No	198	79.8	50	20.2		
Yes	203	68.6	93	31.4		
Depression					98.27	**< 0.001**
None	381	80.9	90	19.1		
Mild	19	31.1	42	68.9		
Moderate or Serious	1	8.3	11	91.7		
Physical symptoms						
Pain	130	65.3	69	34.7	11.39	**0.001**
Shortness of breath	51	60.7	33	39.3	8.66	**0.003**
Insomnia	106	64.2	59	35.8	10.96	**0.001**
Weakness	127	69.8	55	30.2	2.35	0.077
Fatigue	100	71.4	40	28.6	0.57	0.258
Nausea	26	59.1	18	40.9	5.28	**0.02**
Lack of appetite	60	57.1	45	42.9	18.44	**< 0.001**
Constipation	74	68.5	34	31.5	1.88	0.107
Diarrhea	29	74.4	10	25.6	0.01	0.547
Edema	28	75.7	9	24.3	0.08	0.476
Cough	64	71.1	26	28.9	0.38	0.311
Vomiting	11	61.1	8	44.4	2.54	0.095
Fever	6	60.0	4	40.0	0.99	0.253
Bloated	49	71.0	20	29.0	0.30	0.342
Health Self-efficacy Score					65.43	**< 0.001**
High(≥50)	261	87.6	37	12.4		
Low(<50)	140	56.9	106	43.1		

Bold face *p* < 0.05

### Effects of physical symptoms on suicidal ideation

Multivariate logistic regression analysis was performed to examine the effect of each physical symptoms on SI, individually. We focused on the six physical symptoms (pain, shortness of breath, insomnia, nausea, weakness, and lack of appetite) that were identified with significant or marginally significant differences in the earlier comparison between the participants with or without SI. Both insomnia (aOR = 1.84, 95% CI 1.13 to 3.00, *p* = 0.015) and lack of appetite (aOR = 2.14, 95% CI 1.26 to 3.64, *p* = 0.005) were found positively associated with SI after adjusting for socio-demographics, clinical variables, and diagnosed depression in model 3 (Table 3).

**Table 3:** Multivariate Logistic Regression Analyses for Predictive Factors of Suicidal Ideation in Cancer Patients. (*N* = 544)

Characteristics	Model 1				Model 2				Model 3			
	aOR	95CI		*p*	aOR	95CI		*p*	aOR	95%CI		*p*
Physical symptoms												
pain	1.62	1.06	2.47	**0.026**	1.33	0.84	2.08	0.223	1.11	0.69	1.79	0.659
shortness of breath	1.37	0.79	2.36	0.265	1.52	0.85	2.73	0.157	1.47	0.80	2.72	0.218
insomnia	2.10	1.34	3.27	**0.001**	2.00	1.25	3.19	**0.004**	1.84	1.13	3.00	**0.015**
weakness	1.03	0.66	1.61	0.901	0.95	0.59	1.52	0.826	0.88	0.54	1.44	0.603
nausea	1.56	0.76	3.20	0.223	1.20	0.57	2.53	0.636	0.95	0.43	2.10	0.891
lack of appetite	2.51	1.56	4.04	**< 0.001**	2.38	1.43	3.94	**0.001**	2.14	1.26	3.64	**0.005**
Health Self-efficacy Score												
Low(<50)vs. High(≥50)					4.98	3.11	7.97	**< 0.001**	3.51	2.13	5.80	**< 0.001**

Bold face *p* < 0.05

aOR (95CI) Adjusted Odds Ratio (95% Confidence Interval)

Model 1& Model 2: Adjusted for demographic and clinical variables including sex, age, marital status, education attainment, employment, metastasis, stage at diagnosis

Model 3: Additionally adjusted diagnosed depression

### Interaction of health self-efficacy and physical symptoms on suicidal ideation

We also tested the modifying effect of health self-efficacy on the association between physical symptoms and SI. As shown in Table 4, physical symptoms of insomnia and lack of appetite were significantly associated with SI in patients with low levels of health self-efficacy. According to a difference in OR between high and low health self-efficacy, two-way interaction terms of health self-efficacy×each physical symptom were entered and tested individually. In model 3, low health self-efficacy showed a marginally significant exaggerating effect on the association between pain and SI (aOR = 2.77, 95% CI 0.99 to 7.74, *p* = 0.053) when adjusted for all the confounding variables.

**Table 4:** Association between physical symptoms, health self-efficacy, and suicidal ideation (*N* = 544)

Physical symptoms and self-efficacy	Model 1				Model 2				Model 3			
	aOR	95CI		*p*	aOR	95CI		*p*	aOR	95%CI		*p*
High-level of health SE (score ≥ 50)												
Pain	0.68	0.29	1.56	0.358	0.62	0.26	1.48	0.279	0.60	0.24	1.45	0.252
Shortness of breath	1.29	0.48	3.49	0.617	1.41	0.51	3.91	0.508	1.39	0.50	3.88	0.533
Insomnia	2.04	0.91	4.58	0.083	2.01	0.87	4.64	0.102	1.87	0.79	4.44	0.063
Weakness	0.78	0.34	1.81	0.563	0.83	0.35	2.00	0.679	0.82	0.34	1.98	0.661
Nausea	0.53	0.06	4.75	0.572	0.37	0.04	3.49	0.382	0.73	0.03	3.29	0.344
Lack of appetite	2.35	0.99	5.59	0.052	2.47	1.01	6.04	**0.047**	2.44	0.99	6.00	0.052
Interaction between physial symptoms and health SE^a^												
Health SE x Pain	3.21	1.20	8.59	**0.02**	3.03	1.12	8.15	**0.028**	2.77	0.99	7.74	0.053
Health SE x Shortness of breath	1.73	0.52	5.77	0.372	1.79	0.54	5.97	0.346	1.82	0.52	6.42	0.351
Health SE x Insomnia	0.95	0.37	2.44	0.906	0.98	0.38	2.55	0.968	1.05	0.39	2.81	0.931
Health SE x Weakness	1.60	0.59	4.29	0.355	1.59	0.59	4.30	0.36	1.52	0.54	4.23	0.427
Health SE x Nausea	5.84	0.57	59.61	0.137	6.02	0.60	60.74	0.128	6.24	0.53	73.16	0.145
Health SE x Lack of appetite	1.03	0.37	2.89	0.959	1.07	0.38	3.05	0.895	1.03	0.35	3.01	0.964

*SE* self-efficacy

Bold face *p* < 0.05

aOR (95CI) Adjusted Odds Ratio (95% Confidence Interval)

Model 1: Adjusted for sex, age, marital status, education attainment, employment and all the other physical sym ptoms

Model 2: Additionally adjusted for clinical variables: metastasis, stage at diagnosis

Model 3: Additionally adjusted for diagnosed depression

^a^The interaction between a physical symptom and health self-efficacy (high /low) was tes ted indivi dually

Because the depression-rating scale HDRS-17 also contains items related to the physical symptoms (i.e. sleep, weight loss, and suicide), which may confound the measures, we then performed another set of analyses by using the HDRS-12 scores that excluded the evaluation of the aforementioned physical symptoms in the depression variable. The results agreed with our previous findings, as shown in the Additional file 2 (Model 3 in Appendix Table 3 & Table 4) with the data of insomnia (aOR = 1.75, 95% CI 1.07 to 2.87, *p* = 0.026), lack of appetite (aOR = 2.20, 95% CI 1.29 to 3.74, *p* = 0.004), and low health self-efficacy × pain (aOR = 2.68, 95% CI 0.96 to 7.47, *p* = 0.056).

## Discussion

Overall, our study revealed that the prevalence of SI was 26.3% among Chinese hospitalized cancer patients. Patients with physical symptoms of insomnia or lack of appetite were more likely to report SI, even after adjusted for socio-demographic variables (marital status, being retired), clinical variables (stage at diagnosis, metastasis), and diagnosed depression. Furthermore, our findings suggest a marginally exaggerating effect of low health self-efficacy on pain and SI, after adjusted for all the confounding variables.

In the present study, 26.3% of the hospitalized patients reported SI following cancer diagnosis, which is higher than the previous reports (15.3–18.4%) on the cancer population in Mainland China [25, 40, 41]. Such prevalence is comparable to the previous studies conducted in Spain, the U.S., and Taiwan (22.6–29.5) [4, 13, 15], but higher than that of some Western studies (7.8–17.7%) [14, 17–20] and much lower than that of Japan and South Korea (34.7–71%) [23, 42–44]. The discrepancy between the reported studies and ours may result from the differences in measures and sample composition. The majority of the patients participating in our study were diagnosed with cancers of the digestive system, breast, and lung. Those with stage III and IV cancers accounted for 70% of the total samples. It has been reported that the cancer site and stage at diagnosis are associated with SI [14, 44]. Moreover, the scale utilized in our study includes both passive and active SI as the outcome. The passive SI refers to the death ideation as a wish to die or preference to die and the active SI refers to thoughts of killing oneself [45]; when both are included in the analysis, it may show a higher prevalence.

To the best of our knowledge, this is the first study to examine the associations of physical symptoms, health self-efficacy and SI, and their interactions on SI among cancer patients. In line with our hypothesis, we found that patients with specific physical symptoms (i.e. lack of appetite, insomnia) were more likely to report SI. Lack of appetite was about twice as likely to be associated with SI after adjusted for socio-demographic and clinical characteristics. Choi et al. also found physical symptoms were significantly associated with SI in stomach cancer patients [23]. Previous studies showed that patients with difficulties in vital function, e.g. eating, experienced more helplessness/hopelessness and were therefore at higher risk of SI [24, 44]. Our observed association between insomnia and SI is also biologically plausible. Sleeping loss causes various endocrine and immunological changes and studies have shown that dysfunction of serotonin (5-hydroxytriptamine) plays a significant role in suicide [46]. Insufficient sleep negatively impacts cognitive function resulting in poor judgment and deficits in impulse control, which might contribute to SI and suicidal behaviors [47]. Moreover, insomnia is not only a diagnostic criterion of depression but also strongly associated with co-occurring mental disorders [48] that increase the risk for suicidality.

The results support our second hypothesis, indicating that low health self-efficacy has a marginally significant exaggerating effect on the association between pain and SI, after adjusting for socio-demographic, clinical, and depression variables. The finding agrees with previous studies, in which patients with comparable levels of pain tend to have less depressive symptoms if they have higher health self-efficacy [49]. Health self-efficacy reflects an individual’s perceived ability to cope with stress and confidence in overcoming challenges to their health [29, 30]. There is evidence that cancer patients with perceived higher health self-efficacy reported lower psychological distress and higher quality of life [31]. In contrast, patients with low health self-efficacy may prematurely terminate coping efforts, consider escaping from the distressing situations, and think of suicide.

There are some limitations in our study. First, our sample is from the oncology settings of two general hospitals in northeast China, so the results may limit generalization to outpatient settings, cancer-specialized hospitals, or other regions of China. Second, the study respondents may have not reported SI due to stigma or embarrassment about suicide behaviors, thus the prevalence of SI reported in our study might be an underestimation. Third, the patients who refused to participate or failed to complete the interview may experience severe physical or emotional distress, which would result in a selection bias as we did not compare the socio-demographics between the participants who completed the survey and those who dropped out. Fourth, our study assessed the physical symptoms over the 2 days prior to the interview, which probably included the occasional or coincidental symptoms that might not be persistently associated with cancer. Fifth, instead of using the continuous HRSD-17 scores, our study used a cut-off score to dichotomize the variable of depression. Sixth, apart from depressive disorders, we did not identify other mental disorders in analyzing the predictors for SI. Finally, a cross-sectional study is not able to determine a cause-effect relationship. Future longitudinal research is needed to explore whether health self-efficacy interventions reduce the incidence of SI and further risks of suicide, e.g. future intent or prior suicide attempts.

One in four hospitalized cancer patients in our study reported SI, indicating that high risk of suicidality remains an issue for this population in China. The present study provides valuable potential implications with respect to the care of cancer patients:1) Paying attention to physical symptoms, especially insomnia and lack of appetite, would help with the early identification of SI and thus prevent suicide; 2) Actively managing the physical symptoms via medical and psychological treatments would decrease the risks of suicide; 3) Promoting a sense of health self-efficacy could play a key role in suicide prevention, particularly for the cancer patients presenting with pain.

## Conclusions

The present study revealed significant associations between physical symptoms (i.e. insomnia, lack of appetite) and SI in cancer patients. Furthermore, health self-efficacy plays a modifying role in the relationship of pain and SI among Chinese hospitalized patients with cancer. Paying attention to these physical symptoms and promoting the sense of health self-efficacy could be helpful for healthcare professionals to detect high-risk patients for suicide early and thus enable timely interventions.

## Supplementary Information

**Additional file 1.**

**Additional file 2: **Table 1 Multiple logistic regression analysis with the backward method in cancer patients. Table 2 Correlations between main variables (*n* = 544). Table 3. Multivariate Logistic Regression Analyses for Predictive Factors of Suicidal Ideation in Cancer Patients. (*N* = 544). Table 4. Association between physical symptoms, health self-efficacy, and suicidal ideation (*N* = 544).

## Data Availability

Data can be gained from the corresponding author.

## Abbreviations

AOR: Adjusted odds ratio
CI: Confidence intervals
SI: Suicidal ideation
YES: Yale Evaluation of Suicidality
MQOL: McGill Quality of Life Questionnaire
SUPPH: Strategies Used by People to Promote Health
HDRS: Hamilton Depression Rating Scale
SE: Self-efficacy
