# Supplementary material for: A cross-sectional study on associations of physical symptoms, health self-efficacy, and suicidal ideation among Chinese hospitalized cancer patients
Source: BMC Psychiatry. 2020 Nov 19;20:544. doi: 10.1186/s12888-020-02945-x (PMC7678141; doi:10.1186/s12888-020-02945-x)
Supplement: Supplementary file 1 — Additional file 1. [file 12888_2020_2945_MOESM1_ESM.docx]

| Often when people deal with serious illnesses, it affects their will to live. We will now ask you questions about your will to live in relation to your current stressors. | | | | | | |
| --- | --- | --- | --- | --- | --- | --- |
| **YALE EVALUATION OF SUICIDALITY SCALE (YES): A New Scale to Assess Vulnerability to Suicide** | | | | | | |
| **(BECK-KOVACS SCALE FOR SUICIDAL IDEATION, MODIFIED)** | | | | | | |
| D1. | In light of current circumstances, how strong would you say your wish to live has been? | | | |  | |
|  |  | | | Strong | | - 1 |
|  |  | | | Moderate | | - 2 |
|  |  | | | Weak | | - 3 |
|  |  | | | Have none | | - 4 |
|  |  | | | REF | | - 7 |
|  |  | | | DK | | - 8 |
|  |  | | |  | |  |
| D2. | In light of your circumstances, how strong has your wish to die been? | | | |  | |
|  |  | | Strong | | | - 1 |
|  |  | | Moderate | | | - 2 |
|  |  | | Weak | | | - 3 |
|  |  | | Have none | | | - 4 |
|  |  | | REF | | | - 7 |
|  |  | | DK | | | - 8 |
|  |  |  | | |  | |
|  |  |  | | |  | |
|  |  |  | | |  | |
| D3. | In light of your current circumstances, have you ever had thoughts of killing yourself? | | | |  | |
|  |  | | No | | | - 1 |
|  |  | | Possibly | | | - 2 |
|  |  | | Yes | | | - 3 |
|  |  | | REF | | | - 7 |
|  |  | | DK | | | - 8 |
|  |  |  | | |  | |
| D4. | In light of your current circumstances, which of the following best describes your feelings about living versus dying? | | | |  | |
|  |  | | Living outweighs dying | | | - 1 |
|  |  | | About equal | | | - 2 |
|  |  | | Dying outweighs living | | | - 3 |
|  |  | | REF | | | - 7 |
|  |  | | DK | | | - 8 |

***If the responses to questions D1 through D4 are 1, 4, 1, 1, respectively, skip the remainder of the YES.***

|  |  |  | | |  | |
| --- | --- | --- | --- | --- | --- | --- |
| D5. | In light of your current circumstances, have you actively wished to make a suicide attempt? | | | |  | |
|  |  | | No | | | - 1 |
|  |  | | Possibly | | | - 2 |
|  |  | | Yes | | | - 3 |
|  |  | | REF | | | - 7 |
|  |  | | DK | | | - 8 |
|  |  | | | |  | |
| D13. | Have you actively planned a suicide attempt? | | | |  | |
|  |  | No | | | | - 1 |
|  |  | Considered; details not finished | | | | - 2 |
|  |  | Yes | | | | - 3 |
|  |  | REF | | | | - 7 |
|  |  | DK | | | | - 8 |
|  |  | | | |  | |
| D17. | Have you attempted suicide in the past? | | | |  | |
|  |  | | No | | | - 1 |
|  |  | | Possibly | | | - 2 |
|  |  | | Yes | | | - 3 |
|  |  | | REF | | | - 7 |
|  |  | | DK | | | - 8 |
|  |  |  | | |  | |
| Please indicate whether you agree or disagree with the following statements. | | | | | | |
|  |  | | |  | |  |
|  |  | | | **Yes** | | **No** |
|  |  | | |  | |  |
| B4. | When things are going badly, I am helped by knowing they can’t stay that way forever. | | | 1 | | 2 |
|  |  | | |  | |  |
| C18. | I am too stable to kill myself. | | | 1 | | 2 |
|  |  | | |  | |  |
| C57. | I believe I am unable to adjust to or cope with my problems. | | | 1 | | 2 |
|  |  | | |  | |  |
| C27. | I would not kill myself because my family needs me and depends on me. | | | 1 | | 2 |
|  | [Interviewer, If caregiver has no suicidal ideation state, If I were feeling like ending my life, one of the reasons I wouldn’t, would be because my family depends upon me and needs me.] | | |  | |  |
|  |  | | |  | |  |
| C33. | I would not kill myself because I am afraid of the actual “act” of killing myself (the pain, blood, violence). | | | 1 | | 2 |
|  | [Interviewer, If caregiver has no suicidal ideation state, If I were feeling like ending my life, one of the reasons I wouldn’t, would be because I would be afraid of the actual act of killing myself (the pain, blood, violence).] | | |  | |  |
|  |  | | |  | |  |
| C45. | I would not kill myself because I consider it morally wrong. | | | 1 | | 2 |
|  | [Interviewer, If caregiver has no suicidal ideation state, If I were feeling like ending my life, one of the reasons I wouldn’t, would be because I would consider it morally wrong.] | | |  | |  |
|  |  | | |  | |  |
| C62. | If I felt like a burden to my family, it would affect my will to live. | | | 1 | | 2 |
|  |  | | |  | |  |
|  |  | | |  | |  |
|  |  | | |  | |  |
| D14. | Do you have access to firearms? | | | |  | |
|  |  | | | Yes | | - 1 |
|  |  | | | No | | - 2 |
|  |  | | | REF | | - 7 |
|  |  |  | | |  | |
| D22. | Has a family member ever tried to commit suicide? | | | |  | |
|  |  | | No | | | - 1 |
|  |  | | Possibly | | | - 2 |
|  |  | | Yes | | | - 3 |
|  |  | | REF | | | - 7 |
|  |  | | DK | | | - 8 |
